# Yolk Sac Elements in Tumors Derived from Pluripotent Stem Cells: Borrowing Knowledge from Human Germ Cell Tumors

**DOI:** 10.3390/ijms26136464

**Published:** 2025-07-04

**Authors:** Marnix van Soest, Joaquin Montilla-Rojo, Thomas F. Eleveld, Leendert H. J. Looijenga, Daniela C. F. Salvatori

**Affiliations:** 1 Anatomy and Physiology, Department Clinical Sciences, Faculty of Veterinary Medicine, Utrecht University, 3584 CL Utrecht, The Netherlands; 2 Princess Máxima Center for Pediatric Oncology, 3584 CS Utrecht, The Netherlands; 3 Department of Pathology, University Medical Center Utrecht, 3508 GA Utrecht, The Netherlands; 4 Centre for Animal-Free Biomedical Translation, 3584 CS Utrecht, The Netherlands

**Keywords:** pluripotent stem cells (PSC), yolk sac tumor (YST), yolk sac elements, PSC-based therapies, safety assessment, malignancy, human germ cell tumor (hGCT), teratoma assay

## Abstract

Pluripotent stem cell (PSC)-based therapies are currently in clinical trials. However, one of the main safety concerns includes the potential for cancer formation of the PSC-derived products. Currently, the teratoma in vivo assay is accepted by regulatory agencies for identifying whether PSCs have the potential to become malignant. Yolk sac elements (YSE) are one of the elements that could arise from PSC. Whereas the other malignant element, embryonal carcinoma, is thoroughly studied, this is not the case for YSE. Therefore, more research is needed to assess the nature of YSE. We propose that it is imperative to include the formation of YSE in the safety assessment of PSC due to their close resemblance to the clinical entity of yolk sac tumor (YST), a human malignant germ cell tumor (hGCT). In this review, we extrapolate knowledge from YST to better understand YSE derived from PSC. We demonstrate that both share a similar morphology and that the same characteristic immunohistochemical markers can be used for their identification. We discuss the risk these tumors pose, thereby touching upon genetic abnormalities and gene expression that characterize them, as well as possible disease mechanisms. Integrating the molecular and immunohistochemical markers identified in this review into future research will help to better address the potential malignancy associated with PSC.

## 1. Introduction

The discovery of induced pluripotent stem cells (iPSC) in 2007 accelerated research into pluripotent stem cell (PSC)-based therapies for disease treatment, with currently over a hundred clinical trials with regulatory approval [1,2]. However, safety concerns regarding the potential for cancer formation after transplantation of PSC-derived therapies remain largely unresolved, hampering the approval of therapies [3,4].

### 1.1. Safety Concerns Regarding PSC-Derived Therapies

The major safety aspect in the development of PSC-based therapies is the similarity between PSCs and malignant cells, posing a possible risk of cancer formation after transplantation [4,5]. Somatic cells used to generate iPSCs may harbor pre-existing genetic abnormalities or acquire them during in vitro culture; these abnormalities are often similar to those observed in human cancers, highlighting the risk of leading to malignancy in vivo [3,6,7,8]. For example, it has recently been described that feeder-free culture of PSC can drive specific genetic changes [9]. The commonly observed genetic abnormalities can be chromosomal aberrations as well as single-nucleotide variants [5,7,10,11]. For instance, PSC can acquire dominant negative *TP53* mutations, which are seen, because of their relation to cancer formation, as a major concern [10,12,13]. For these reasons, the recently published guidelines for human stem cell research recommend periodically testing for chromosomal abnormalities and single-nucleotide variants [14].

### 1.2. The In Vivo Teratoma Assay: The Current Method to Asses Malignant Potential of PSC

A direct link between genetic abnormalities and functional malignancy, meaning the risk of cancer formation from the PSC used, remains to be fully clarified [3]. To assess the potential of malignant tumor formation in PSC, current guidelines recommend performing the in vivo teratoma assay [14].

The in vivo teratoma assay has been the main method to confirm the pluripotency of stem cells [15,16]. The assay involves injecting stem cells, which are expected to be pluripotent, across multiple anatomical sites of immunocompromised mice. Places of injection are subcutaneous, intramuscular, under the kidney capsule, or intratesticular. At these anatomical sites, stem cells may develop tumors. The cell line can be defined as functionally pluripotent when the resulting xenograft exhibits characteristics of a teratoma, i.e., the presence of differentiated cells from all three germ layers: ectoderm (e.g., nerve and skin), mesoderm (e.g., bone, cartilage, and muscle), and endoderm (e.g., liver and gut) [15].

In certain cases, the teratomas that develop may also include elements described by histopathology as embryonal carcinoma (EC) and/or undifferentiated cells [17]. When these elements are present, the tumor has historically been classified as ‘teratocarcinoma’, a term derived from cancer research in mice, indicating the injected stem cell to have a malignant potential [17].

Nowadays, pluripotency can be assessed in vitro using bioinformatic assays such as the PluriTest and ScoreCard [18]. PluriTest is based on two decades of transcriptomic analyses on pluripotent cell lines, and it compares the transcriptome of a line with a large reference of other lines [19]. ScoreCard analyses the expression of germ layer-specific genes after directed or spontaneous differentiation of a cell line [20]. Based on this expression, ScoreCard provides quantitative insight into the differentiation potential of the cell line [20,21].

International guidelines require the use of the teratoma assay to evaluate the safety of PSCs and PSC-derived products that are intended for clinical use [14,18]. To date, the teratoma assay remains the only test capable of simultaneously assessing the pluripotency and malignancy potential of PSCs [18]. However, the teratoma assay still harbors substantial limitations—being time-intensive, costly, and ethically controversial due to the use of laboratory animals and the assays has to date never been standardized, including the anatomical site of injection (see above) [22].

The readout of the teratoma assay is qualitative and based on histopathology [22]. There have also been attempts to make the readout more quantitative in the form of the TeratoScore assay [18,23]. The features related to malignancy are mostly associated with the presence of EC [15,17]. Also, the presence of elements such as yolk sac, immature neuroectoderm, and undifferentiated cells is seen as a cause of concern regarding malignancy potential [18].

The term teratocarcinoma indicates that the stem cells used are malignant and not safe for injection into humans [17]. Stem cell biologists prefer to use this (simplified) term out of practical reasons, as it distinguishes teratoma, only containing tissue from the three germ layers, from a tumor, which contains teratoma and malignant elements. In human pathology, the term teratocarcinoma is considered obsolete, and similar tumors follow an updated classification [15,24,25].

### 1.3. Borrowing Knowledge of Human Germ Cell Tumors for a Better Understanding of PSC-Derived Tumors

In human pathology, teratomas are classified as belonging to the entity of germ cell tumors (GCT) [26]. GCTs are cancers that are derived from the stem cells of the early embryo and the germ line and are localized in the gonads and along the midline of the body (Box 1) [27]. Teratoma is considered a benign GCT in prepubertal patients when it contains only differentiated mature tissues coming from at least one of the 3 germ layers [27]. However, when other GCT varieties, such as yolk sac tumor (YST), are found besides the teratoma, then the tumor is considered malignant. In post-pubertal GCT patients, the teratoma can be found pure or mixed with other GCT, such as EC or YST, and it is in all cases considered malignant. GCTs are useful in the investigation of malignancy of stem cells because they are seen as a neoplastic counterpart of normal development and pluripotency [26]. For instance, research into pluripotency has been performed for a long time on EC as well as embryonic stem cells, where the EC is seen as the malignant counterpart. [28,29]. As previously mentioned, EC is not the only potential malignant element found in the teratoma assay; elements of the yolk sac can also be found [18]. The teratoma derived from PSC mostly resembles prepubertal (type I) GCT [30]. Specifically, in prepubertal patients, the presence of yolk sac either as a pure tumor or in combination with teratoma is related to malignancy [27]. Therefore, the safety assessment of PSC must also consider the malignant potential occurring from the yolk sac [18].

This review aims to clarify the nature and malignant potential of yolk sacs associated with teratomas derived from PSC, from here on named yolk sac elements (YSE), by extrapolating from the research on GCT. Here, we examine the histopathological characteristics of human GCTs and tumors that are experimentally derived from human pluripotent stem cells, with an emphasis on YSE. Subsequently, we give a comprehensive description of the identification of YSE, discuss the risk YSE poses and describe potential disease mechanisms.

Box 1 **Glossary of terms used in this review.**

**Germ Cell Tumor (GCT)**. A tumor that is derived from (primordial) germ cells of various stages of maturation. These tumors typically occur in the ovaries or testicles but can also appear around the midline of the body and may have various histological subvariants.

 

**Prepubertal (Type I)**. GCTs predominantly occurring in prepubertal patients. Mostly with the histology of pure (benign) teratoma but can also occur as pure yolk sac tumor or in the combination of teratoma and/or yolk sac tumor (being malignant).

 

**Post-pubertal (Type II)**. GCTs occurring in post-pubertal patients. These malignant tumors have an origin in a precursor lesion called germ cell neoplasia in situ and can occur in a more diverse array of histological subtypes, being subdivided into seminoma-like and nonseminoma (embryonal carcinoma, yolk sac tumor, choriocarcinoma, teratoma, and mixed).

 

**Teratoma**. A type of GCT that consists of tissues from all three embryonic germ layers.

**Teratocarcinoma**. A term used by stem cell biologists when they transplant PSC in mice. This term is used to define a malignant form of teratoma that has developed in the mouse, where the teratoma also contains embryonal carcinoma-like cells and/or elements of yolk sac. This term is not used anymore by pathologists, being replaced with terms such as (mixed) nonseminoma; however, this term is still used by stem cell biologists in the context of the in vivo teratoma assay.

 

**Yolk Sac (YS)**. A normal structure that is found during human and animal development. It consists of a membranous structure, forming a sac, which is attached to the embryo, providing early nourishment and is found to contribute to the formation of the gastrointestinal tract and blood cells.

 

**Yolk Sac Tumor (YST)**. A malignant germ cell tumor that resembles a structure similar to the yolk sac. Can be either prepubertal (type I) or post-pubertal (type II).

 

**Yolk Sac Element (YSE)**. In this review, this term is used to specifically define elements that resemble a structure similar to yolk sac that are found in teratoma derived from xenografted pluripotent stem cells.

 

**Embryonal Carcinoma (EC)**. A malignant germ cell tumor, appearing as undifferentiated cells and sharing similarities with embryonic stem cells. Can also be found mixed with other types of GCT. EC has been described in teratoma derived from xenografted PSC.

## 2. Histopathological Similarities Between GCT and Tumors Derived from PSC Relevant to Malignancy

### 2.1. Histopathology of Human GCT

In human pathology, teratoma is described as a tumor that can be composed of tissues that resemble the three embryonic germ layers—ectoderm, mesoderm, and endoderm—in a disorderly manner [15,26]. According to the WHO classification of 2022, teratoma can be subdivided into teratoma prepubertal-type (I) and teratoma post-pubertal-type (II) (non-seminomatous GCT), depending on both the age of the patient and the cell of origin [25], represented by the (epi) genetic constitution [27]. Teratoma can contain both mature tissues (mature teratoma) and immature tissues (immature teratoma) [15].

EC is characterized by sheets, glands, and papillary structures composed of primitive epithelial cells with pleomorphic nuclei and containing little to extensive cytoplasm [26,31]. EC can be found in the form of a pure tumor or in combination with other GCT elements; in this case, the neoplasia is described as a mixed GCT [26]. Mixed GCTs are tumors that are composed of a combination of multiple different GCTs, for example, a mixed GCT composed of both EC and teratoma or a combination of EC and YST [26]. It is hypothesized that the EC cells, being the malignant stem cells of the mixed GCT, can differentiate into both somatic and extra-embryonic tissues, such as YST [26]. The mixed GCT can also contain elements of seminoma (called dysgerminoma in the ovary and germinoma in the brain), another type of GCT mimicking embryonic germ cells. Nonetheless, these will not be touched upon in this review since seminomatous-like elements are never found to be associated with teratomas derived from PSC [27].

Human YST can have a vast array of morphologies as described in the literature [32]. The main feature is that the morphology contains cells and structures similar to those found in embryonic or fetal yolk sacs. Most frequently, these structures resemble a spider web-like network of cells. Another key identifying feature is the presence of Schiller-Duval bodies, which are structures with a vascular core lined by cuboidal or columnar endodermal epithelium [32]. YST can be pure tumors with the whole tumor resembling the yolk sac, or they can be part of mixed GCT containing teratoma, EC, or other GCT types [26].

### 2.2. Histopathology of Tumors Derived from PSC Compared to GCT and the Risks YSE Pose

**Figure 1:**
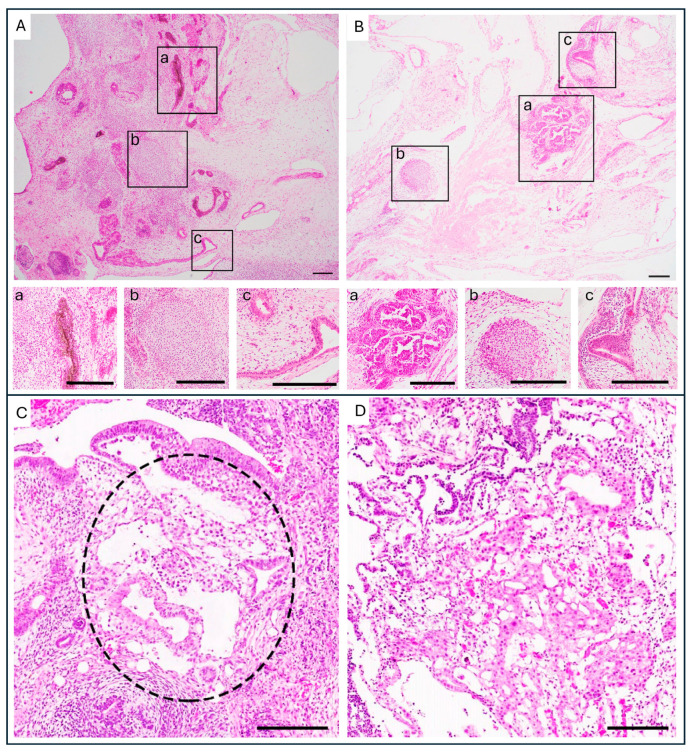
Histopathological features of PSC-derived teratoma. (**A**) PSC-derived teratoma showing the three germ layers: (**a**) ectoderm (pigmented epithelium), (**b**) mesoderm (early cartilage) and (**c**) endoderm (gut epithelial tissue). (**B**) Different parts of PSC-derived teratoma (**A**) containing: (**a**) embryonal carcinoma (**b**) mesoderm (early cartilage) and (**c**) endoderm (gut epithelial tissue). (**C**) Example of loosely structured yolk sac elements indicated by the circle inside of a PSC-derived teratoma. (**D**) Example of loosely structured yolk sac elements inside of a PSC-derived teratoma. Images derived from the Salvatori Lab (teratoma and embryonal carcinoma) and adapted from [33] or adapted according to the Creative Commons CC-BY from [15] (yolk sac element). Scale bars, 200 µm.

Structures resembling EC and YST can be recognized in tumors derived from PSC after transplantation in mice, based on the same morphological patterns seen in human GCTs [15,33] (Figure 1). Yolk sac elements (YSE) have been found in teratomas after injection of PSC in immunodeficient mice [15,18,34,35]. However, it is not yet understood whether they fully resemble the malignant YST or rather the benign embryonic yolk sac.

Histological evidence on its own cannot conclusively determine if the presence of YSE indicates the malignant potential of the PSC. Its similarity to YST; however, raises considerable concern [18]. YST are malignant tumors—meaning, in this context, a higher risk of metastatic behavior and treatment resistance. For example, prepubertal (Type I) GCTs can be purely composed of (benign) teratoma. However, it can also be found mixed with YST, or it can consist purely out of YST. In prepubertal (Type I) GCTs, YST is therefore the only malignant element [27]. In line, the presence of YST caused a higher chance of relapse after chemotherapy in a cohort of prepubertal GCT patients [36]. The presence of YST also leads to a poorer outcome in post-pubertal patients, as shown in an investigation of 615 patients with testicular GCT, where the presence of YST in the primary cancer was significantly associated with a detrimental prognosis [37]. YST leads to a worse prognosis compared to other GCTs over all age groups, which has recently also been found when retrospectively studying over 27,000 patients [38]. Furthermore, a high concentration of alpha-fetoprotein (AFP) (>10,000 ng/mL), a marker directly related to YST, in liquid biopsies from patients with metastatic GCT is a poor prognostic indicator, according to the International Germ Cell Consensus Classification Group (IGCCG) [39,40].

### 2.3. Immunohistochemistry of YST and YSE in Teratomas Derived from PSC

To distinguish YST from other GCTs (for instance, EC), OCT4 is used as the baseline staining [41,42]. OCT4 is universally positive in EC and seminoma-like GCT, but is also universally negative in YST [42]. To further differentiate YST from other GCT that are not EC or seminoma, YST-specific stainings are used [42]. AFP and Glypican-3 (GPC3) are the markers recommended by the World Health Organization for the diagnosis of YST [24]. Over time, new markers for YST have been introduced (Table 1). GPC3 is more sensitive than AFP in immunohistochemistry, staining all occurring morphologies of YST, whereas AFP stains 85% of YST [42]. GPC3 is, however, found to be less specific, staining immature elements in teratoma (37%) as well as a small percentage of EC (5%) [42,43]. The marker Zinc finger and BTB domain-containing protein 16 (ZBTB16, also known as PLZF) is highly sensitive for YST, staining all morphologies of YST, as well as specific, staining negative for all other GCT, including teratoma and EC [44]. Sal-like protein 4 (SALL4) is a highly sensitive marker for YST, more sensitive than GPC3 and AFP [45,46,47]. However, SALL4 also stains EC and seminoma-like cells, and can stain focally in immature parts of teratoma, as well as sparsely in enteric-type glands, making it not specific [45]. Other YST markers CDX2, HNF1β, and FOXA2 are less specific, also staining parts of teratoma with endodermal differentiation (intestine) [48,49,50].

For the identification of YSE in tumors derived from PSC, the same markers used for YST are informative. The Standards of the International Society for Stem Cell Research (ISSCR) recommend using CDX2, GPC3, AFP, and SALL4 as markers for YSE in tumors developing from PSC [14]. Also, other YST markers, such as ZBTB16 and FOXA2, might be used to identify YSE. Due to the complexity of PSC-derived teratomas, it is difficult to rely on only one single marker. In fact, some antibodies stain differentiated elements of teratoma; for instance, endodermal differentiated tissue (intestine, pancreatic acini) [48,49,50]. A large study, performed by the ISCI in 2018, showed a good precedent by using a combination of multiple markers to identify malignant elements derived from PSC [18]. The researchers tested approaches for the identification of pluripotency and malignancy of PSC, and they used the combination of OCT4 (EC vs. YSE and teratoma), SALL4 (positive for YSE and EC), and ZBTB16 (YSE vs. EC and teratoma) [18]. Pathological assessment in combination with this staining panel can be regarded as the baseline to identify YSE and EC in tumors derived from PSC. Further research with testing staining panels for specifically targeting YSE or identifying new, more specific markers would help refine the optimal staining panel for these elements.

## 3. Genomics and Pathways of Malignancy Found in Yolk Sac Tumor

### 3.1. Chromosomal Aberrations Found in YST

Chromosomal aberrations are frequently observed in GCTs, including YST, both Type I (prepubertal) and II (post-pubertal). In general, prepubertal YSTs are often aneuploid, possibly near diploid [27]. Prepubertal teratoma, on the other hand, the other most commonly found GCT in prepubertal patients, is diploid and benign, suggesting that the presence of chromosomal aberrations may contribute to a more malignant phenotype [27]. In general, pre- and post-pubertal YST have similar chromosomal aberrations; the gain of the short arm of chromosome 12 (12p) is, however, more commonly observed in post-pubertal YST. This chromosomal aberration is also present in almost all other post-pubertal GCTs. Gain of 1q is also commonly found in GCT [27,55,56]. Amplification of the long arm of chromosome 20 (20q) is a chromosomal aberration often found in prepubertal YST [27]. Unique chromosomal changes that are frequently found in post-pubertal and prepubertal YST and hardly detected in the other GCT are the loss of 1p and 6q [27,55,56]. Specific gain of chromosomal regions can also be related to drug resistance. Recently, 3p25.3 was identified as a predictor of poor prognosis in post-pubertal type GCTs, i.e., associated with cisplatin resistance [57]. Interestingly, although the cisplatin-resistant effect of a gain of 3p25.3 was observed in all post-pubertal type GCT, the presence of the gain occurred significantly more often in patients with YST [57,58].

It is unknown whether the YSE found in teratoma derived from PSC harbors genetic aberrations and can thus be regarded as malignant. The parallel between PSC and GCT favors a hypothesis regarding a shared mechanism of genomic instability and potential malignant transformation. Specifically, the amplifications of 20q and 1q are found both in PSC as well as in a large group of cancers (including YST) [3,7,8,9,27]. It is suggested that YST specifically could develop these genetic aberrations because of its extraembryonic nature [27]. In mice studies, blastocysts generated from both polyploid murine ESC and diploid murine ESC show extraembryonic lineages originating from the polyploid cells, while the embryonic lineages come from the diploid cells [59,60]. This suggests that differentiation towards extraembryonic lineages, thereby not contributing to the somatic lineages, might be an escape mechanism for cells with genetic abnormalities [27]. A recent paper [61] showed that aneuploid murine ESCs but not isogenic diploid murine ESCs could metastasize after xenotransplantation and teratoma formation, again showing the potential importance of genetic aberrations. Interestingly, the chromosomal regions of the aneuploid chromosomes studied by Xiao et al. seem non-random, but overlap with regions related to growth advantage in PSCs and malignancy in GCTs [61,62]. Future research would need to be performed to show the exact influence of genomic instability on PSC safety.

### 3.2. YST Gene Expression

Some authors have expressed a significant need for highly sensitive bioinformatic assays to aid in the identification of the malignant potential of PSC lines [3,18]. The current bioinformatic assays are not yet able to perform such an identification of malignant transformation of PSC [3,18]. For example, the TeratoScore assay, a bioinformatic assay based on mRNA expression, was found to be unable to identify malignant transformation of PSC [18,23]. A potentially overlooked fact regarding the identification of malignant transformation of PSC by using gene expression data is that yolk sac (tumor) has a differential gene expression compared to EC, which are both malignant elements that could arise from PSC [51,63,64]. By studying multiple GCT types, YST was found to exhibit distinctive gene expression from explicitly EC, with upregulation of genes related to differentiation—endodermal genes and factors related to WNT, BMP, and GATA signaling pathways—and downregulation of pluripotency-related genes [51,63,64]. In addition to differences in mRNA expression, YST show also a differential DNA methylation pattern compared to other GCTs [64,65]. This differential gene expression would make the use of only one gene set and/or methylation profile to identify all potential malignant elements derived from PSC, such as been tried previously [18], more difficult. Therefore, considering the findings in GCTs, it might be beneficial for future bioinformatic assays based on gene expression or DNA methylation to include YST-specific gene expression and methylation, or even score YSE presence separately from EC.

### 3.3. Mechanisms Related to Malignancy: YST and WNT Signaling

YST are found to have a higher expression of genes related to the WNT pathway compared to other GCT [51,64,66]. Additionally, it has been known for a long time that YSTs have active WNT signaling; however, it is not determined if this signaling is causative in the transformation to YST or if it reflects a feature of the corresponding tissue [27,67]. Nevertheless, there are recent studies that relate WNT signaling to YST, including one demonstrating that the WNT pathway was found to be activated in GCT by activating mutations [66]. Interestingly, the mutations were found either in pure YST or in mixed GCT with a YST component [66]. Importantly, in the same study, the inhibition of the WNT pathway with inhibitors leads to a reduced proliferation of explicitly YST lines, which indicates an important role for the WNT pathway in the disease mechanism of YST [66].

In GCT, and in YST specifically, the WNT pathway might be activated via multiple triggers outside of activating mutations. Firstly, the miRNA cluster 371–373 was found to upregulate the WNT pathway by downregulation of DKK1, a protein that inhibits the WNT pathway [68]. This miRNA 371–373 cluster is proposed as a potential biomarker, which can be observed in the blood, for malignant GCTs, including EC and YST but excluding teratoma [69,70,71,72,73,74]. Notably, in the light of PSC safety, this miRNA cluster is also upregulated in malignant teratomas derived from PSC, which contain elements of EC and YSE [35].

Secondly, the WNT pathway can be activated by the recruitment of WNT ligands to the cell membrane by the YST marker GPC3. Several studies have proposed that GPC3 functions as a mediator, facilitating the attraction of WNT ligands to the cell surface and subsequently activating the pathway [75,76]. As discussed previously, GPC3 has emerged as a significant marker for YST, demonstrating upregulated expression at both mRNA and protein levels [51,54]. Therefore, GPC3 might mediate a YST-specific activation of WNT. This has been proposed as a disease mechanism in both hepatocellular carcinoma as well as osteosarcoma, where GPC3 is also highly expressed [77,78,79]. In osteosarcoma, GPC3 has been shown to colocalize with active β-catenin on the cell membrane, and treatment with a specific anti-GPC3 antibody led to less β-catenin localization [78]. In hepatocellular carcinoma, both antibody-mediated targeting of GPC3 and chimeric antigen receptor T-cell (CAR-T) therapy directed against GPC3 have demonstrated efficacy in suppressing WNT signaling [76,80]. These studies indicate that the GPC3-mediated WNT signaling could be a mechanism of disease development. Future studies need to be performed to see if this WNT-related disease mechanism is also relevant in YST.

Lastly, in YST it was found that the *APC* promotor is often hypermethylated [64,81]. The accumulation of β-catenin, and consequently the activation of WNT signaling, was linked to hypermethylation of *APC* [81]. Whether the formation of YST causes *APC* hypermethylation or vice versa needs further investigation. However, this specific hypermethylation indicates the importance of the WNT pathway in YST. It is not yet known what the methylation status is of *APC* is in YSE found in teratoma derived from PSC. Its investigation would be an interesting future avenue of study.

WNT signaling is crucial for maintaining pluripotency in stem cells [82,83]. However, as we have shown, when we extrapolate from the knowledge of YST, dysregulation of WNT signaling might contribute to either the formation of YSE or to the potential disease mechanism of YSE, meaning that sustained WNT signaling might be necessary for YSE to survive. Identifying aberrant WNT signaling might aid in investigating the potential malignancy of PSC. However, the exact role of WNT signaling, the miRNA cluster 371–373, GPC3 and APC in the context of PSC-derived tumors remains to be investigated.

## 4. Discussion and Conclusions

Before bringing PSC-based therapies to the clinic, the field needs to make sure that these therapies are safe. Allogenic stem cell therapies show potential to create new off-the-shelf therapies for multiple diseases, of which some are already in clinical trials [1]. Important with these off-the-shelf therapies is that the original PSC source is safe, since an overlooked safety risk in such a line could have consequences for a potentially substantial number of patients receiving the therapy.

**Figure 2:**
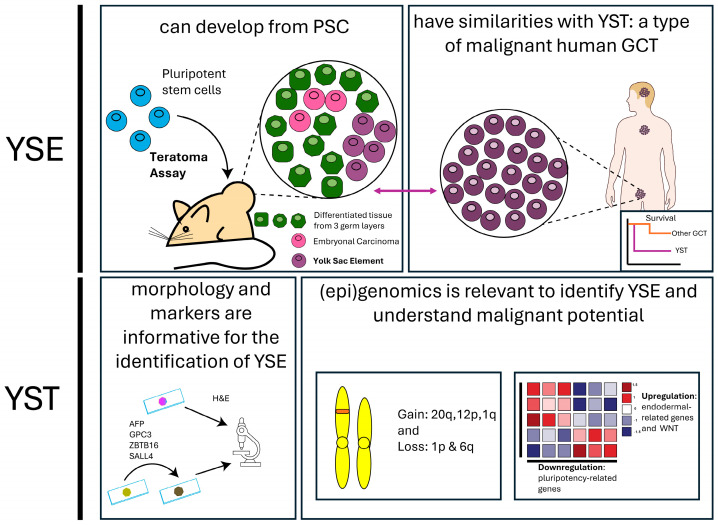
Graphical overview: Yolk Sac Elements (YSE) can develop from pluripotent stem cells when injected into mice. These YSE have similarities with Yolk Sac Tumor (YST) which is a malignant type of human Germ Cell Tumor. This review explained how YSE can be recognized based on markers and morphology. YST have a distinctive genomic profile which is also shown to be informative in understanding YSE. Abbreviations: YSE (Yolk Sac Element), YST (Yolk Sac Tumor), PSC (Pluripotent Stem Cells). Source male illustration: NIAID Visual & Medical Arts. (7 October 2024). Human Male Outline Type I. NIAID NIH BIOART Source. https://bioart.niaid.nih.gov/bioart/233 (accessed on 19 May 2025).

PSC safety can be hampered by multiple factors, of which the propensity of tumor formation is critical. While the risk of the formation of EC from PSC is better understood, there is still a lack of understanding of what the formation of extraembryonic tissue in the form of YSE would entail for the safety of PSC. Here, we showed that the presence of yolk sac in the form of YST in GCT is associated with poor prognosis. YST is malignant in both prepubertal GCT as well as post-pubertal GCT. At this moment, little is known about YSE, but the knowledge regarding YST might be extrapolated to YSE (Figure 2). Here, we provided an overview of the characteristics of YST, highlighting markers, gene signatures, and disease mechanisms. This information could be used to recognize YSE formation better and understand its malignant potential.

Importantly, this information should be evaluated and validated regarding YSE in the future. For instance, markers used to identify YST could be further evaluated against YSE by taking along a broader staining panel in their identification. Furthermore, in prepubertal GCTs, aneuploidy is associated with malignant potential. Investigation and detection of chromosomal aberrations in YSE would support the hypothesis of their malignant potential. We have also shown that YST has a distinct gene expression profile. Advanced sequencing methods, like single-cell RNA sequencing of tumors from xenografted PSC that contain YSE, could offer a more comprehensive view of the genes expressed in these elements. By using this technique, it could be possible to contrast the gene expression profiles of YSE with the known YST expression, again highlighting their malignant potential. Lastly, the WNT signaling pathway seems to be important in the formation and survival of YST. Future experiments could investigate which role the WNT pathway plays in the formation of YSE as well as their survival. In addition to the genomics and pathways discussed in this review, metabolic features and post-translational modifications may also be relevant to understanding the malignant potential of YSE. As research regarding YSE is sparse and its malignant potential is currently based on histopathological features, investigation of these metabolic and post-translational modification features in YSE could be a topic for future research.

In summary, the identification and characterization of YSE is vital for enhancing the safety assessment of stem cell products. By integrating the molecular markers and gene signatures identified in this review into future research, we can better address the potential malignancy associated with PSC.

## Figures and Tables

**Table 1:** List of markers that can be used in immunostainings to identify YST and YSE (in teratoma derived after injection of PSC in mice) and related references.

Marker	Cellular Localization	Expression in YSE	Expression in YST
AFP	Cytoplasmic	Reported [15,32,34]	[31,42,45,46,47,49,50,51,52]
CDX2	Nuclear	Not reported	[46,48,53]
FOXA2	Nuclear	Not reported	[50,51]
GPC3	Cell membrane/cytoplasmic	Reported [15,35]	[42,43,45,46,47,50,51,52,54]
HNF1β	Nuclear	Not reported	[49]
SALL4	Nuclear	Reported [18]	[42,45,47,51]
ZBTB16	Nuclear	Reported [18]	[44,52]

## Abbreviations

The following abbreviations are used in this manuscript:

PSC	Pluripotent Stem Cell
iPSC	Induced Pluripotent Stem Cell
GCT	Germ Cell Tumor
EC	Embryonal Carcinoma
YST	Yolk Sac Tumor
YSE	Yolk Sac Element
WHO	World Health Organization
AFP	Alpha-Fetoprotein
OCT4	Octamer-binding Transcription factor 4
GPC3	Glypican-3
ZBTB16	Zinc finger and BTB domain-containing protein 16
SALL4	Sal-like protein 4
HNF1β	Hepatocyte Nuclear Factor 1 homeobox B
FOXA2	Forkhead Box Protein A2
ISCI	International Stem Cell Initiative
BMP	Bone Morphogenetic Protein
APC	Adenomatous Polyposis Coli
